# Extraction, Purification, and Hydrolysis Behavior of Apigenin-7-O-Glucoside from *Chrysanthemum Morifolium* Tea

**DOI:** 10.3390/molecules23112933

**Published:** 2018-11-09

**Authors:** Yuxiao Wang, Zhenzhen Xu, Yuqi Huang, Xin Wen, Yue Wu, Yuhan Zhao, Yuanying Ni

**Affiliations:** 1 College of Food Science and Nutritional Engineering, China Agricultural University, Beijing 100083, China; wangg5211@163.com (Y.W.); yqh18207731071@163.com (Y.H.); 952823491@163.com (X.W.); wy1102742656@163.com (Y.W.); 13120164842@163.com (Y.Z.); 2 National Engineering Research Center for Fruit and Vegetable Processing, Beijing 100083, China; 3 Key Laboratory of Fruit and Vegetable Processing, Ministry of Agriculture, Beijing 100083, China; 4 Institute of Quality Standard & Testing Technology for Agro-Products, Chinese Academy of Agricultural Sciences, Beijing 100083, China; xuzhenzhen@caas.cn

**Keywords:** *Chrysanthemum morifolium*, apigenin-7-O-glucoside, purification, hydrolysis

## Abstract

Apigenin-7-O-glucoside is an active phenolic compound in Asteraceae flowers and possesses remarkable therapeutic applications. However, its high price and low abundance in plants limit its use, meanwhile it would hydrolyze in the purification process. In this study, apigenin-7-O-glucoside extracted with ultrasound and purified with preparative HPLC from *Chrysanthemum morifolium* ‘Huangju’ was investigated, as well as its hydrolysis behavior and bioactivities. The optimized extraction conditions were: solid/liquid ratio: 1:20, extraction time: 35 min, temperature: 50 °C, and ultrasound power: 350 W. The content of apigenin-7-O-glucoside was up to 16.04 mg/g. Apigenin-7-O-glucoside was then purified with preparative HPLC from the extract, and confirmed by Q-TOF/MS. Apigenin-7-O-glucoside was partially hydrolyzed in acidic condition, and the hydrolysis rate depended on the pH value and temperature. The antioxidant activity increased as a result of the hydrolysis process. This study provided a green and effective way to obtain apigenin-7-O-glucoside and would be beneficial for further investigations into nutritional and functional aspects apigenin-7-O-glucoside and other glycosides.

## 1. Introduction

Tea brewed from chrysanthemum flowers has been extensively utilized for centuries. It is reported that *Chrysanthemum morifolium* could reduce hyperactivity of the liver, and improve eyesight. These healing benefits of *Chrysanthemum morifolium* are closely related to the composition and content of phenolic compounds [1,2,3]. Apigenin-7-O-glucoside is one of the active phenolic compounds in chrysanthemum flowers [4,5], which is reported to possess remarkable antispasmodic, anti-inflammatory, antioxidant, and anticarcinogenic properties [2,5,6,7,8]. However, its high price of about US $498 per 10 mg limits the use of apigenin-7-O-glucoside.

In addition to its high price, its low abundance in plants also limits its use. Elmastas et al. [9] analyzed changes in the content of apigenin-7-O-glucoside obtained from *Rosa* species over the harvest period using HPLC-diode array detection (DAD), and the content varied from 4.13 to 24.42 mg/kg (dry basis) between different species at different harvest times. Apigenin-7-O-glucoside was low abundant in *Rosa* species. Uehara et al. [10] reported the presence of luteolin-7-O-glucoside, luteolin-7-O-glucuronide, luteolin-7-O-xylosylglucoside, luteolin, apigenin, eriodictyol, and naringenin in *C. arcticum* ssp. *arcticum* and *yezoense* and related *Chrysanthemum* species using an HPLC-photodiode array. However, there was no report of the presence of apigenin-7-O-glucoside, which indicated that it was not the main compound in the above species. From the corresponding liquid chromatograms, the contents of apigenin-7-O-glucoside were found to be very low by the area normalization method, which demonstrated that the analyzed materials were not suitable for extracting apigenin-7-O-glucoside.

Several types of Asteraceae plants are described in the literature for their content in apigenin-7-O-glucoside [11]. In a companion study [4], an investigation of phenolic compounds in various Asteraceae plants using ultrahigh-performance liquid chromatography–ultraviolet spectroscopy–quadrupole time-of-flight mass spectrometry (UHPLC-UV-Q-TOF/MS) was reported. In comparison with Roman and German chamomile, the content of apigenin-7-O-glucoside was much higher in Chinese chrysanthemum flowers, which indicates that Chinese chrysanthemum flowers are good resources for the extraction of apigenin-7-O-glucoside. However, the active constituents of these cultivars have not been studied or characterized with respect to the content of apigenin-7-O-glucoside, which is a serious problem when deciding which kind of *Chrysanthemum morifolium* flowers to select for extraction and purification. Sensitive, efficient, and selective methods for the purification of apigenin-7-O-glucoside from chrysanthemums are also scarce in the scientific literature.

Meanwhile, we found that apigenin-7-O-glucoside would hydrolyze in the purification process by preparative HPLC. The formic acid was necessary to be added for improving separation and peak performance. There was no literature about its hydrolytic behavior and antioxidant activity. Because of this, our work was conducted to (1) screen new cultivars of *Chrysanthemum morifolium*, (2) optimize the extraction and purification conditions, and (3) investigate the hydrolytic behavior and antioxidant activities. This study provides a green and effective approach for obtaining apigenin-7-O-glucoside and will be helpful for research on the development of further investigations into nutritional and functional aspects.

## 2. Results and Discussion

### 2.1. Screening the Appropriate Chrysanthemum Morifolium Cultivar

**Figure 1:**
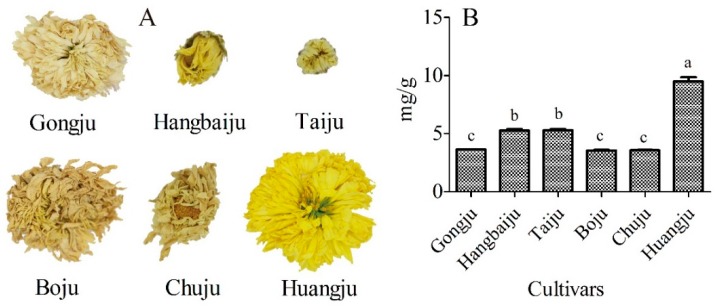
Screening the appropriate cultivar for the extraction and purification of apigenin-7-O-glucoside. **A**. The flowers of *Chrysanthemum morifolium* ‘Gongju’, *C. morifolium* ‘Hangbaiju’, *C. morifolium* ‘Tai’, *C. morifolium* ‘Boju’, *C. morifolium* ‘Chuju’, and *C. morifolium* ‘Huangju’. **B**. Content of apigenin-7-O-glucoside in different cultivars. Means indicated by different letters differed significantly with a value of *p* < 0.01.

As shown in Figure 1A, the flowers were used to screen the appropriate cultivar for the purification of apigenin-7-O-glucoside, including *Chrysanthemum morifolium* ‘Gongju’, *C. morifolium* ‘Hangbaiju’, *C. morifolium* ‘Tai’, *C. morifolium* ‘Boju’, *C. morifolium* ‘Chuju’, and *C. morifolium* ‘Huangju’. As shown in Figure 1B, *C. morifolium* ‘Huangju’ had a higher content of apigenin-7-O-glucoside than the other cultivars. Avula et al. [4] found that the content of apigenin-7-O-glucoside in Roman and German chamomile was lower than that in Chinese chrysanthemums using UHPLC-UV-Q-TOF/MS (see supplementary Figure 1). Moreover, Uehara et al. [10] analyzed some Japanese cultivars, such as *Chrysanthemum arcticum* ssp. *arcticum* and *yezoense*, but detected no apigenin-7-O-glucoside in a study using HPLC and MS. The content of apigenin-7-O-glucoside varied between different cultivars. *C. morifolium* ‘Huangju’ was therefore screened as the raw material to optimize the ultrasound conditions.

### 2.2. Optimization of Ultrasound Conditions

**Figure 2:**
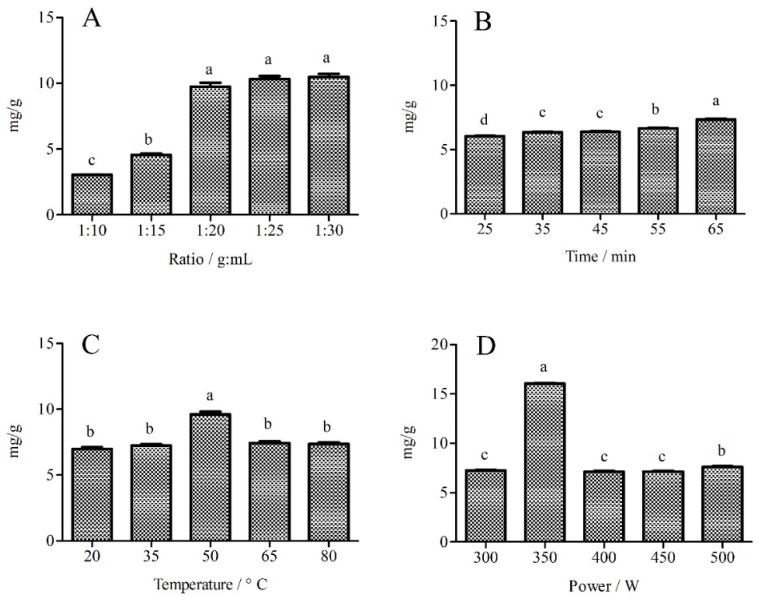
Optimization of the extraction conditions assisted with ultrasound. Results using: (**A**) solid/liquid ratios (g/mL) from 1:10 to 1:30; (**B**) extraction times from 25 to 65 min; (**C**) temperatures from 25 to 80 °C; and (**D**) ultrasound power from 300 to 500 W. Means indicated by different letters differed significantly with a value of *p* < 0.01.

Extraction of natural products assisted by ultrasound has been widely employed in food processing. The solid/liquid ratio, extraction time, temperature, and ultrasound power were optimized in the extraction process. As shown in Figure 2A, the content of apigenin-7-O-glucoside in the extract increased with the solid/liquid ratio. Owing to the structure of apigenin-7-O-glucoside, the introduction of the glucoside moiety increases its polarity and water solubility. Water was therefore used as an environment-friendly reagent to substitute organic solvents in the extraction process. A ratio of 1:20 (g/mL) was selected because the content increased without significant difference (*p* < 0.01) at higher ratios.

As shown in Figure 2B, there was no significant difference between extraction times of 35 and 45 min (*p* < 0.01). Even though the content of apigenin-7-O-glucoside continued to increase with the extraction time, other factors, such as energy consumption, should also be taken into account. As shown in Figure 2C, the content of apigenin-7-O-glucoside reached a high value at a temperature of 50 °C, while the content decreased at temperatures of 65 and 80 °C. In contrast to microwave extraction, the temperature would not increase rapidly in extraction assisted by ultrasound. There was an interesting phenomenon in that the content at an ultrasound power of 350 W was higher than those at other power values, as shown in Figure 2D. This phenomenon probably occurred because a decrease in power increased the pulse interval. Pan et al. [12] reported that pulse ultrasound was superior to continuous ultrasound with respect to energy consumption, reduction of the extraction time, and antioxidant yields. The conditions were optimized as follows: a solid/liquid ratio of 1:20, an extraction time of 35 min, a temperature of 50 °C and an ultrasound power of 350 W. The content of apigenin-7-O-glucoside was up to 16.04 mg/g from *C. morifolium* ‘Huangju’ under the optimized extraction conditions. The content in *C. morifolium* ‘Huangju’ was much higher than that reported in *Rosa* species [9].

### 2.3. Analysis and Purification Of Apigenin-7-O-Glucoside from C. Morifolium ‘Huangju’

**Figure 3:**
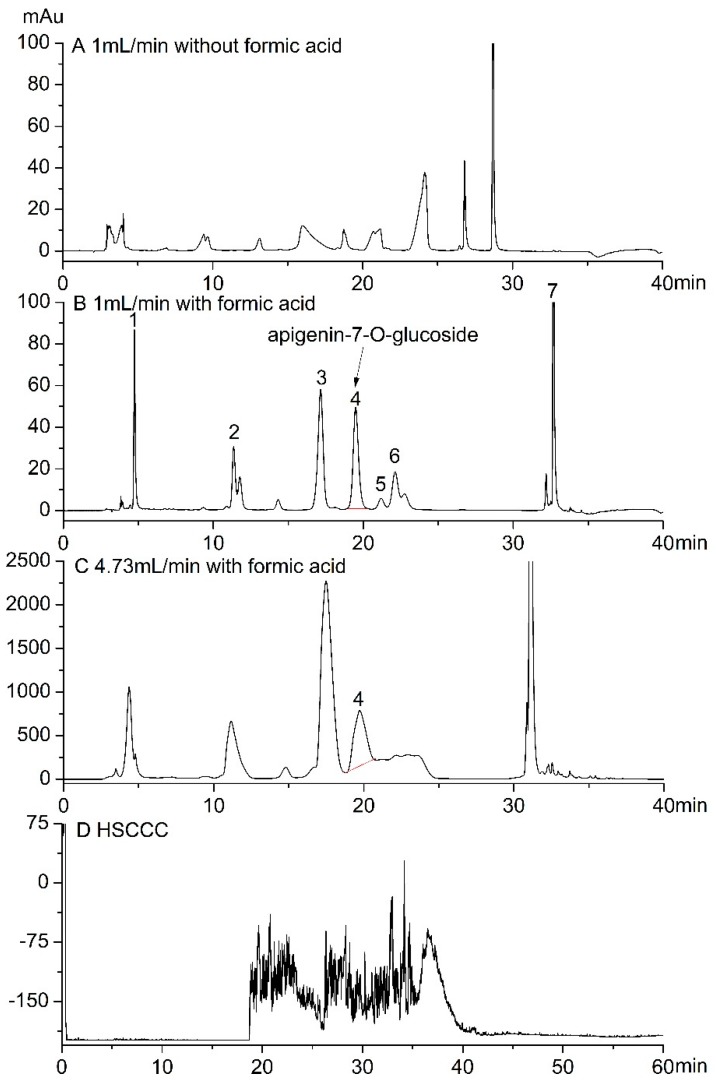
Analysis and purification of apigenin-7-O-glucoside from *C. morifolium* ‘Huangju’. 1: chlorogenic acid, 2: luteolin-7-O-glucoside, 3: 3,5-dicaffeoylquinic acid, 4: apigenin-7-O-glucoside, 5: apigenin-7-O-glucuronide, 6: luteolin-7-O-6”-malonylglucoside, 7: apigenin. (**A**) 10 μL of 10 mg/mL chrysanthemum extract at a flow rate of 1 mL/min without formic acid. (**B**) 20 μL of 10 mg/mL chrysanthemum extract at a flow rate of 1 mL/min with formic acid. (**C**) 400 μL of 100 mg/mL chrysanthemum extract at a flow rate of 4.73 mL/min with formic acid with preparative HPLC. (**D**) 400 μL of 100 mg/mL chrysanthemum extract with HSCCC.

HPLC analysis of the aqueous extract revealed the presence of multiple compounds in *C. morifolium* ‘Huangju’ (Figure 3). The following components were identified by Q-TOF/MS: chlorogenic acid, luteolin-7-O-glucoside, 3,5-dicaffeoylquinic acid, apigenin-7-O-glucoside, apigenin-7-O-glucuronide, luteolin-7-O-6”-malonylglucoside, and apigenin (Table 1). Except apigenin, the other compounds had high polarity. Without formic acid, a high ratio of water was needed to separate these compounds Figure 3A. Without formic acid, the phase A (water) and phase B (acetonitrile) was optimized as follows: 0–14 min with 2-2% B, 14–15 min with 2–100% B, 15–30 min with 10–100% B, 30–31 min from 100% to 2% B, and 32–42 min with 2-2% B. Even the ratio of water was set from 2:98 (water:acetonitrile, *v*/*v*), the chromatographic peak was still poor. The introduction of formic acid increased the retention and separation effect Figure 3B. Therefore, the gradient elution solvents consisted of phase A (water with 0.1% formic acid) and phase B (acetonitrile) was further optimized as follows: 0–25 min with 20-20% B, 25–30 min with 20–50% B, 30–35 min with 50–100% B, 35–37 min from 100% to 20% B, and 37–42 min with 20-20% B. From Figure 3B, apigenin-7-O-glucoside displayed a retention time of 19.575 min. The components of chrysanthemums are complex. Other literatures have also introduced an analysis of apigenin-7-O-glucoside. Lin et al. [13] analyzed apigenin-7-O-glucoside from *C. morifolium* (‘Hangbaiju’ or ‘Taiju’) using HPLC-DAD. However, in the chromatograms apigenin-7-O-glucoside could not be separated from diosmetin-7-O-rutinoside and 3,5-dicaffeoylquinic acid.

As for separation and purification, the most common methods were column chromatography, preparative high-performance liquid chromatography (Pre HPLC), high-speed countercurrent chromatography (HSCCC) [14], and thin-layer chromatography (TLC) [15]. In consideration of separation effect, the Pre HPLC and HSCCC would be more suitable for purification. The separation effect of HSCCC (Figure 3D) and preparative HPLC (Figure 3B) was compared. A two-phase solvent system composed of ethyl acetate-butyl alcohol-water (4:1:5, *v*/*v*/*v*) was used for the polar compounds from *C. morifolium* ‘Huangju’. HSCCC could separate the components in a high load volume but was not suitable for separating complex components such as those present in *C. morifolium* ‘Huangju’ (Figure 3D). The development of a HSCCC method might be difficult owing to both the variability of the phenolic compounds present in the phenolic fraction and differences in their concentration and polarity [4]. The purification of apigenin-7-O-glucoside was performed via preparative HPLC according to the optimized HPLC analysis, and the flow rate was increased to 4.73 mL/min (Figure 3C).

The purified apigenin-7-O-glucoside has the formula C_21_H_20_O_10_ and a mass of 431.09914 [M − H]^−^ (see supplementary Figure 2). Regarding the fragmentation pattern, this compound has a major ion with *m*/*z* 431.09906 [M − H]^−^ and another secondary ion with *m*/*z* 269.04463 [M − H]^−^. By comparison with MS and MS/MS data in the literature [13,16,17,18,19], the purified compound was identified as apigenin-7-O-glucoside.

### 2.4. Hydrolysis Behavior

**Figure 4:**
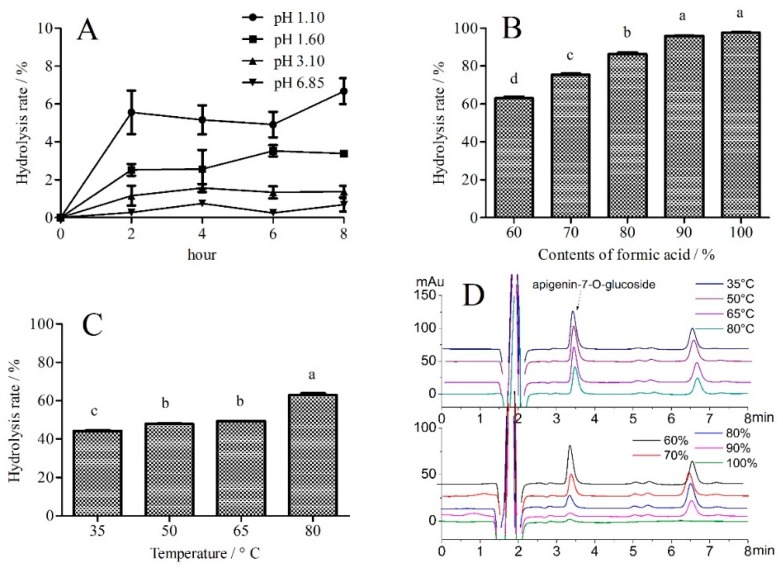
Hydrolysis behavior of apigenin-7-O-glucoside. (**A**) pH values from 1.10 to 6.85 at 80 °C; (**B**) content of formic acid from 60 to 100% at 80 °C; (**C**) temperatures from 35 to 80 °C with 60% formic acid; and (**D**) HPLC spectrograms about the contents of formic acid and temperature. Means indicated by different letters differed significantly with a value of *p* < 0.01.

As shown in Figure 4A, the hydrolysis rate of apigenin-7-O-glucoside reached a maximum at a pH of 1.10. One interesting feature was that the hydrolysis rate largely remained unchanged after 2 h. In theory, an excess amount of formic acid was present in the hydrolysis system. Hydrolysis of apigenin-7-O-glucoside could also be used to simulate digestion by gastric juice, i.e., at a pH of 1.60 [20], because Figure 4A demonstrates that hydrolysis of apigenin-7-O-glucoside would also occur in the stomach.

The effect of different contents of formic acid was further investigated, and the results showed that apigenin-7-O-glucoside was partially hydrolyzed in acidic conditions, as shown in Figure 4B,D, and completely hydrolyzed when the content of formic acid reached 90%. When the content of formic acid reached 100%, no HPLC peak was detected at a retention time of 6.5 min, which indicated that the structure was destroyed, see Figure 4D. As shown in Figure 4C, the hydrolysis rate continued to increase with an increase in temperature, which could explain why the content of apigenin-7-O-glucoside decreased at temperatures of 65 and 80 °C in the ultrasonic extraction process.

### 2.5. Antioxidant Activities

The antioxidant activities of apigenin-7-O-glucoside, apigenin, and glucose were assessed by comparing their EC_50_ values in radical scavenging and metal chelation tests. As shown in Table 2, neither apigenin-7-O-glucoside, nor apigenin, nor glucose exhibited antioxidant activity in 2,2-diphenyl-1-picrylhydrazyl (DPPH) radical scavenging tests, or ferrous ion chelation assays. Even the positive controls—namely, butylated hydroxytoluene (BHT), ascorbic acid, and rutin—did not display any level of ferrous ion chelating activity.

The EC_50_ values of apigenin-7-O-glucoside and apigenin in 2,2′-azinobis(3-ethylbenzothiazoline-6-sulfonic acid) (ABTS) radical scavenging tests were 5.49 and 0.68 mg/mL, respectively (Table 2), which constituted a significant difference in EC_50_ values (*p* < 0.01). The ABTS radical scavenging ability of apigenin was not significantly (*p* < 0.01) different from those of BHT, ascorbic acid, and rutin, whereas the ABTS radical scavenging ability of apigenin-7-O-glucoside was significantly (*p* < 0.01) lower than those of the positive controls. These EC_50_ values showed that the antioxidant activity of apigenin was higher than that of apigenin-7-O-glucoside, which indicated that the antioxidant activity increased as a result of the hydrolysis process. This raised the question of whether it was apigenin-7-O-glucoside or its hydrolyzate that exhibited the therapeutic effects. Further investigations should be performed to compare the effects of apigenin-7-O-glucoside and its hydrolyzate in relevant therapeutic applications, which would be beneficial for clarifying their functional mechanisms.

## 3. Materials and Methods

### 3.1. Materials

*Chrysanthemum morifolium* ‘Gongju’ and *C. morifolium* ‘Hangbaiju’ were bought from Beijing Tongrentang Health Pharmaceutical Industry Co., Ltd. (Beijing, China). *C. morifolium* ‘Taiju’ was purchased from Beijing Zhang Yiyuan Jinqiao Tea Co., Ltd. (Beijing, China). *C. morifolium* ‘Boju’ was procured from Bozhou Zhongyitang Chinese Medicinal Materials Sales Co., Ltd. (Bozhou, China). *C. morifolium* ‘Chuju’ was bought from Anhui Jutai Chuju Herb Science and Technology Co., Ltd. (Anhui, China). *C. morifolium* ‘Huangju’ was purchased from Huangshan Dingxiangwu Ecological Agriculture Development Co., Ltd. (Huangshan, China). Apigenin-7-O-glucoside, formic acid, and acetonitrile were obtained from Sigma-Aldrich (St Louis, MO, USA). Ultrapure water (18.2 MΩ cm) was further purified using a Milli-Q system (Millipore, Billerica, MA, USA). Unless otherwise mentioned, the chemicals and reagents used in this experiment were of analytical grade.

### 3.2. Sample Preparation

In this study, the flowers were selected as the raw materials, including *Chrysanthemum morifolium* ‘Gongju’, *C. morifolium* ‘Hangbaiju’, *C. morifolium* ‘Tai’, *C. morifolium* ‘Boju’, *C. morifolium* ‘Chuju’, and *C. morifolium* ‘Huangju’. These Chinese chrysanthemum flowers were ground to a powder and screened through a 100-mesh filter. The samples were stored at −80 °C before analysis.

### 3.3. Optimization of Ultrasound Conditions

The solid/liquid ratio (0.1 g: 1 to 3 mL), time (25 to 65 min), temperature (20 to 80 °C), and power (300 to 500 W) were optimized in the ultrasound treatment. Initially, 0.1 g chrysanthemum powder was added to a 10 mL PE tube, and then ultrapure water was added. After being vortexed, the tube was placed in an ultrasonic cleaner (KQ-800DE, Kun Shan Ultrasonic Instruments Co., Ltd., Kunshan, China) for rapid extraction. The solution was centrifuged with a high-speed refrigerated centrifuge (CR 21G III, Hitachi, Chiyoda-Ku, Japan) at 10,000 rpm for 1 min. The extraction volume decreased as the chrysanthemum powder absorbed water. Approximately, 0.1 g chrysanthemum powder absorbed 0.8 mL water in the extraction experiments. The resulting extracts were filtered through a 0.22 μm nylon membrane before analysis and purification by preparative HPLC. Before purification, the solution was extracted under the optimized ultrasound conditions and further dried with a rotary evaporator (R100, Buchi, Flawil, Switzerland) coupled with a pump (MVP 10 Basic, IKA, Guangzhou, China).

### 3.4. Analysis and Purification of Apigenin-7-O-Glucoside by Preparative HPLC

Apigenin-7-O-glucoside was analyzed using a preparative HPLC system (Agela Technologies, Tianjin, China) equipped with two solvent pump modules and an ultraviolet detector coupled to a column oven. The crude extract obtained by ultrasound treatment was analyzed with a Venusil ASB C18 column (250 × 4.6 mm i.d., 5 μm, Agela Technologies, Tianjin, China) at 327 nm. A comparison was made to evaluate the effect of formic acid. The injection volume was 20 μL (10 mg/mL) for analysis and 400 μL (100 mg/mL) for purification. Purification was performed by ramping the flow rate from 1 mL/min to 4.73 mL/min linearly using another Venusil ASB C18 column (250 × 10 mm i.d., 5 μm, Agela Technologies, Tianjin, China).

To compare the separation of HSCCC and preparative HPLC, a TBE-300C high-speed counter-current chromatography (Tauto Biotechnique Company, Shanghai, China) was employed in the present study. A two-phase solvent system composed of ethyl acetate-butyl alcohol-water (4:1:5, *v*/*v*/*v*) was used for the polar compounds. The injection volume was 400 μL (100 mg/mL). The separation process was according to Chen et al. [14]. The apparatus was rotated at 900 rpm, after the hydrodynamic equilibrium was established, the sample was injected into the column. During the separation process, the column temperature was controlled at 25 °C by water-circulating constant temperature implement. The effluent of the column was monitored with a UV detector at 327 nm.

A standard curve (R^2^ = 0.9968) for apigenin-7-O-glucoside was calculated with reference to the content (0.1, 0.25, 0.5, 1, 2.5, or 5 mg/mL) and the area under the respective chromatographic separation curve. After water was added, 0.1 g chrysanthemum powder would absorb water and swell to about 0.8 mL. The content of apigenin-7-O-glucoside in the chrysanthemum samples was calculated using the equation

(1) Content (mg/g)=C×(Vt−Vs)/Mt

where *C* is the concentration (mg/mL) calculated from the standard curve, *Vt* is the total volume (mL) of the extraction solvent, *Vs* is the swelling volume (mL) of the chrysanthemum sample, and *Mt* is the total mass (g) of chrysanthemum added.

### 3.5. Analysis with Q-TOF/MS

MS and MS/MS data were obtained with a Q-TOF liquid chromatography (LC)/MS system (1290 Infinity II, 6545 Q-TOF LC/MS, Agilent, Santa Clara, CA, USA). The conditions were the same as those used for HPLC, and a three-way valve was used to transfer the sample to the injector port. The MS conditions were set as follows: gas temperature 250 °C, drying gas flow 7 L/min, sheath gas temperature 350 °C, sheath gas flow 11 L/min, capillary voltage 3500 V, nozzle voltage 0 V, fragmentor voltage 130 V, skimmer voltage 65 V; MS range 100–3000 *m*/*z*, acquisition rate 4 spectra/s, acquisition time 250 ms/spectrum; MS/MS range 100–3000 *m*/*z*, acquisition rate 3 spectra/s, acquisition time 333.3 ms/spectrum; targeted MS/MS fixed collision energies 10, 20, and 40 eV. The MS data were qualitatively analyzed using MassHunter Workstation software (version B.07.00, Agilent Technologies, Beijing, China) coupled with the METLIN metabolite database supported by Agilent Technologies (Beijing, China).

### 3.6. Hydrolysis Behavior of Apigenin-7-O-Glucoside

Hydrolysis behavior of apigenin-7-O-glucoside was performed in different conditions (pH, time, temperature, and content of formic acid). Briefly, 10 μL apigenin-7-O-glucoside solution (2.5 mg/mL) was mix with 990 μL formic acid solution, and kept in a water bath (35 to 80 °C). The content of apigenin-7-O-glucoside was detected every two hours (n = 3). A UPLC column (Venusil ASB C18, 2.1 × 150 mm i.d., 3 μm, Agela Technologies, China) was used to shorten the analysis time. The analysis condition was set 0.3 mL/min with injection volume of 5 μL, and isocratic elution with water and acetonitrile (75:25, *v*/*v*).

### 3.7. In-Vitro Antioxidant Activities

The changes of antioxidant activities in hydrolysis process were performed by comparing the EC_50_ values of apigenin-7-O-glucoside, apigenin, and glucose. The capacity of ABTS radical scavenging activity, DPPH radical scavenging activity and metal chelating activity were detected by a multimode microplate reader (Spectramax id5, molecular devices). A series concentration was prepared before use. The positive controls, namely, BHT, ascorbic acid, rutin, and EDTA, were used to compare the in vitro antioxidant activities. The in vitro antioxidant activity was calculated according to the equation

(2) $$\mathrm{Activity}\;\left(\%\right)=\frac{A0-A1}{A0}\times 100$$

where *A*0 is the absorbance of the solution without a sample and *A*1 is the absorbance of the solution containing a sample.

#### 3.7.1. ABTS Radical Scavenging Activity

The capacity of ABTS radical scavenging activity was determined according to the previous report [21] with some modifications. 10 mL of 7 mmol/L ABTS were added to 10 mL of 4.95 mmol/L aqueous potassium persulfate, then the mixture was kept for 12 h in dark at room temperature. The solution was then diluted 20 times for further use. The variation in absorbance was detected at 734 nm by mixing 290 μL ABTS solution with 10 μL apigenin-7-O-glucoside, apigenin and glucose, and incubated for 6 min without light.

#### 3.7.2. DPPH Radical Scavenging Activity

The DPPH free radical-scavenging activity was evaluated according to previous study [21,22] with slight modifications. 10 μL of the test sample was added to 290 μL of 0.06 mmol/L solution of DPPH in ethanol. The mixture was mixed and incubated for 30 min in the dark at room temperature, and then the absorbance was determined at 517 nm.

#### 3.7.3. Metal Chelating Activity

Chelating activity was determined using an established method [22] with slight modifications. The samples (100 μL) at various concentrations were mixed with 2 mmol/L FeCl_2_ (50 μL). After 5 min, 5 mmol/L ferrozine (100 μL) was added to the solutions. The mixtures were incubated at room temperature for 10 min and the absorbance was measured at 562 nm.

### 3.8. Statistical Analysis

The results were presented as the mean and with a standard deviation (n = 3). Statistical comparisons were carried out using a least significant difference test of variance (ANOVA) to identify where differences occurred. A statistically significant difference was defined by a value of *p* < 0.01. The analyses were carried out using SAS (version 9.2, SAS Institute, Cary, NC, USA) and GraphPad Prism (version 5.01, GraphPad Software, Inc., La Jolla, CA, USA).

## 4. Conclusions

Because its high price and low abundance limit the exploitation of the remarkable therapeutic properties of apigenin-7-O-glucoside, a method was developed for purifying it from *C. morifolium* flowers. In a screening study, the content of apigenin-7-O-glucoside was higher in *C. morifolium* ‘Huangju’ than in other cultivars. Because of the presence and properties of the glucoside moiety, extraction with water assisted by ultrasound was used as an environmentally-friendly process. The extraction conditions were optimized as follows: a solid/liquid ratio of 1:20, an extraction time of 35 min, a temperature of 50 °C, and an ultrasound power of 350 W. The content of apigenin-7-O-glucoside extracted from *C. morifolium* ‘Huangju’ flowers reached 16.04 mg/g under the optimized extraction conditions. In comparison with other purification methods, pre-HPLC was more suitable for purification. Hydrolysis tests showed that apigenin-7-O-glucoside was partially hydrolyzed in acidic conditions, and the hydrolysis rate depended on the pH value and temperature. Hydrolysis of apigenin-7-O-glucoside would also occur in the stomach. The antioxidant activity increased as a result of the hydrolysis process, which raised the question of whether it was apigenin-7-O-glucoside or its hydrolyzate that exhibited the therapeutic effects. This study has provided a green and effective approach for obtaining apigenin-7-O-glucoside. Moreover, it showed that the hydrolyzate exhibited higher antioxidant activity, which would be helpful for further investigations into nutritional and functional aspects of apigenin-7-O-glucoside and other glycosides.

## Figures and Tables

**Table 1:** Compounds identified from *Chrysanthemum morifolium* ‘Huangju’ by UHPLC-Q-TOF-MS.

No.	RT	Formula	[M – H]^−^	Score	MS/MS	Identification
1	4.833	C16H18O9	353.08785	97.69	191.05644, 248.97382, 112.98560	Chlorogenic acid
2	11.442	C21H20O11	447.09376	87.93	285.03972	Luteolin-7-O-glucoside
3	17.242	C25H24O12	515.12035	95.93	353.08736, 179.03450, 173.04544, 135.04501, 191.05577	3,5-dicaffeoylquinic acid
4	19.575	C21H20O10	431.09920	95.44	268.03810, 269.04341	Apigenin-7-O-Glucoside
5	21.275	C21H18O11	445.07743	79.28	269.04514, 113.02422	Apigenin-7-O-glucuronide
6	22.208	C24H22O14	533.09396	94.85	489.10472, 285.04009	Luteolin-7-O-6”-malonylglucoside
7	32.767	C15H10O5	269.04627	94.96	117.03469, 151.00383, 149.02439	Apigenin

**Table 2:** Antioxidant activities of apigenin-7-O-glucoside, apigenin, and glucose

±	ABTS (EC_50_)	DPPH (EC_50_)	FI (EC_50_)
Apigenin-7-O-glucoside	5.49 ± 0.74 ^a^	/	/
Apigenin	0.68 ± 0.01 ^b^	/	/
Glucose	/	/	/
BHT	0.17 ± 0.00 ^b^	0.41 ± 0.01 ^a^	/
Ascorbic acid	0.12 ± 0.00 ^b^	0.11 ± 0.00 ^b^	/
Rutin	0.52 ± 0.10 ^b^	0.52 ± 0.07 ^a^	/
EDTA	/	/	0.32 ± 0.03

Each value is expressed as the mean ± standard deviation (n = 3). Means with different letters with in a column are significantly different (*p* < 0.01). ABTS and DPPH, effective concentration at which 50% of radicals are scavenged (mg/mL); FI, ferrous ion chelating power; effective concentration at which 50% of ferrous ions are chelated (mg/mL). Positive controls were: BHT, ascorbic acid, rutin, and EDTA. /, no data obtained from the EC_50_ model Y = 100/(1 + 10^((LogEC50-X) * HillSlope)).

## Supplementary Materials

Supplementary File 1
