# Supplementary material for: Extraction, Purification, and Hydrolysis Behavior of Apigenin-7-O-Glucoside from Chrysanthemum Morifolium Tea
Source: Molecules. 2018 Nov 9;23(11):2933. doi: 10.3390/molecules23112933 (PMC6278536; doi:10.3390/molecules23112933)
Supplement: Supplementary File 1 [file molecules-23-02933-s001.zip › Supplementary data.docx]

**Supplementary data**


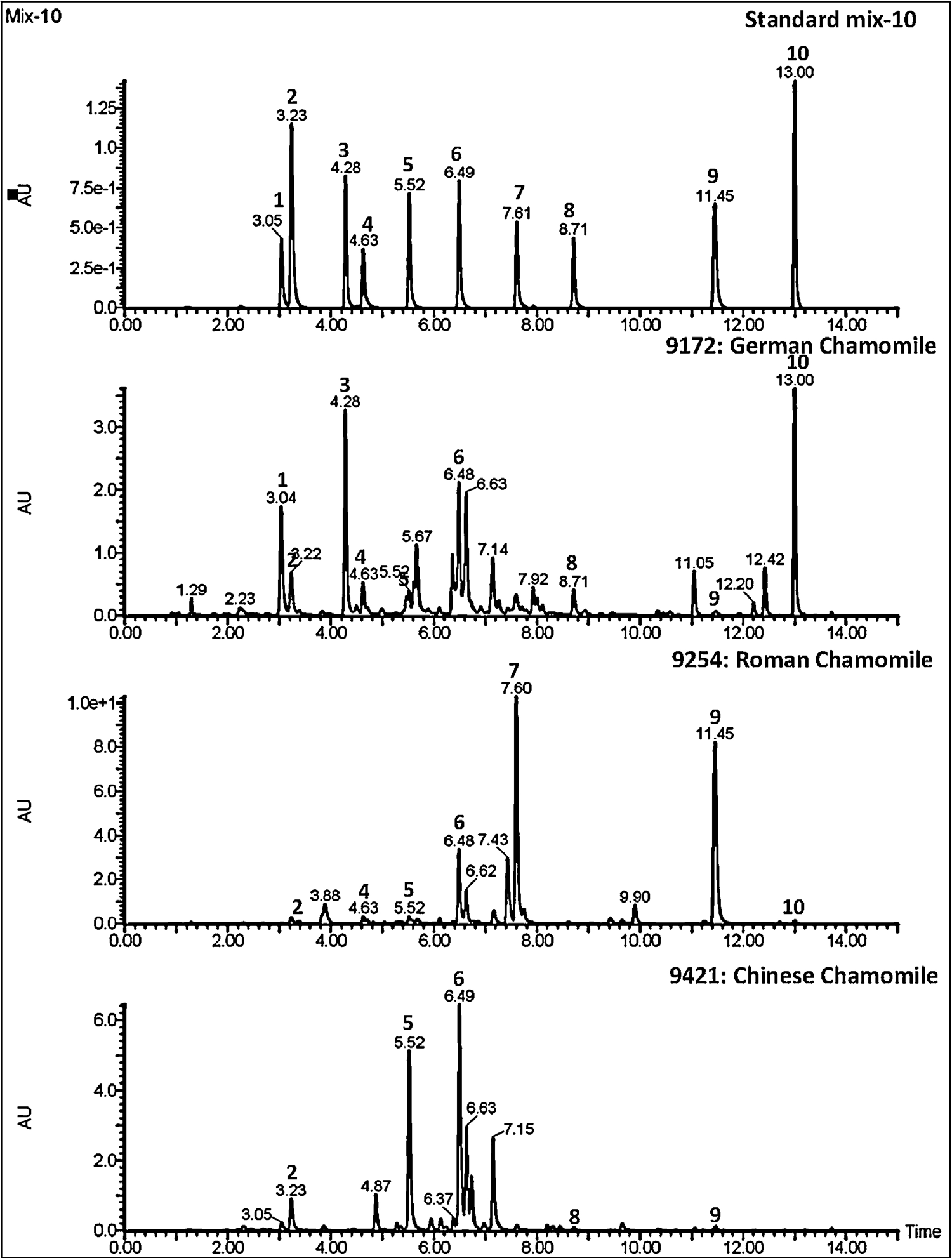


**Figure 1** UHPLC-UV chromatograms of standard mix, German (#9172: *M. recutita*), Roman (#9254: *A. nobilis*) and Chinese chamomile (9421:*C. morifolium*) at 330 nm; apigenin-7-O-β-d-glucoside (6), data from Avula (2014).

**Figure 2** MS and MS/MS spectra of apigenin-7-O-glucoside. (a) MS data for the compound obtained in negative ESI mode. MS/MS data for the compound at collision energies of (b) 10, (c) 20, and (d) 40 eV, respectively.


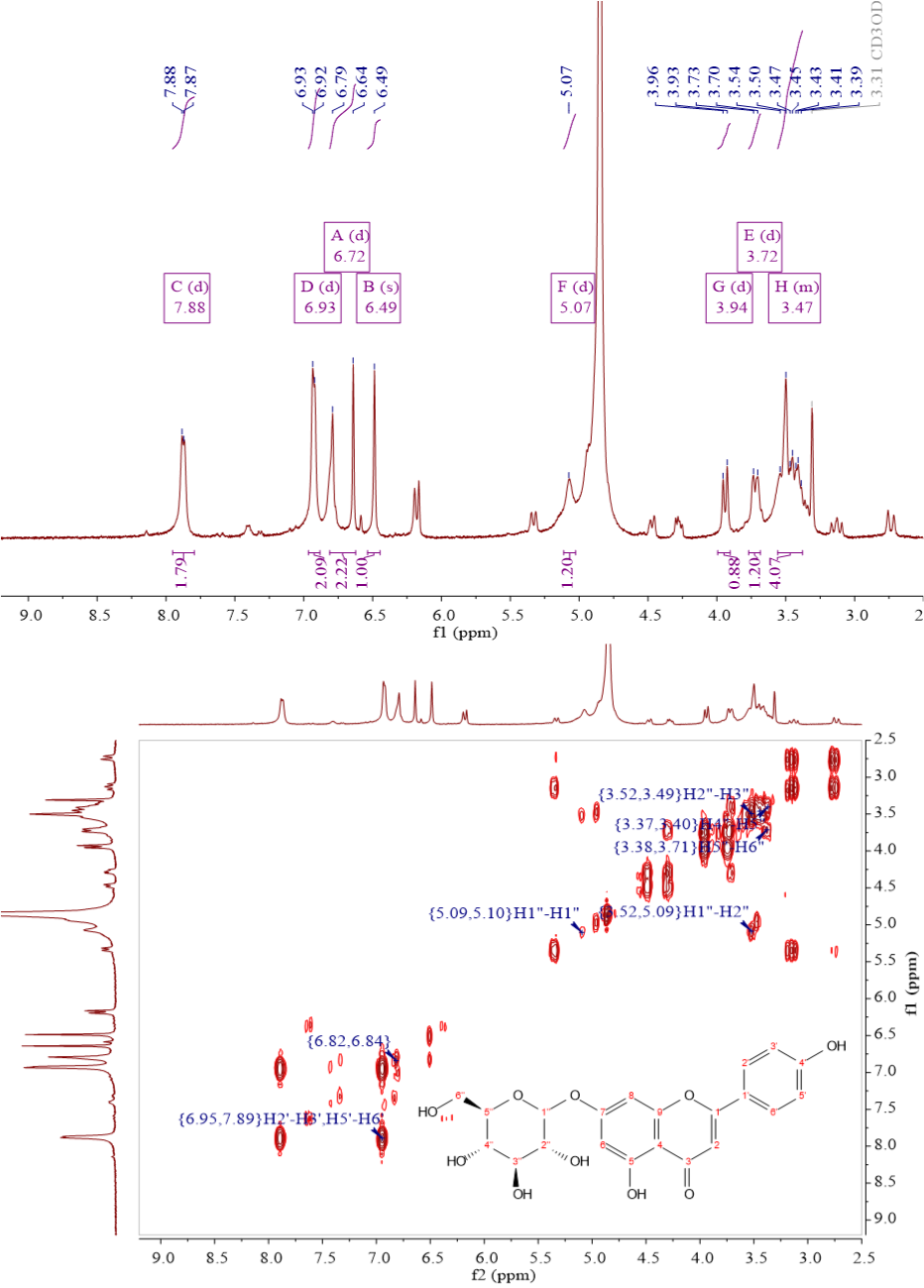


**Figure 3** ^1^H NMR and H-H COSY spectra of purified apigenin-7-O-glucoside.
